# What’s suffering got to do with it? A qualitative study of suffering in the context of Medical Assistance in Dying (MAID)

**DOI:** 10.1186/s12904-021-00869-1

**Published:** 2021-11-11

**Authors:** Barbara Pesut, David Kenneth Wright, Sally Thorne, Margaret I. Hall, Gloria Puurveen, Janet Storch, Madison Huggins

**Affiliations:** 1 School of Nursing, University of British Columbia Okanagan, ARTS 3rd Floor, 1147 Research Road, Kelowna, BC V1V 1V7 Canada; 2 School of Nursing, University of Ottawa, Ottawa, Ontario K1H 8M5 Canada; 3 School of Nursing, University of British Columbia, Vancouver, BC V6T 2B5 Canada; 4 Society of Notaries Public of BC, Chair in Applied Legal Studies, School of Criminology, Simon Fraser University, Surrey, BC V5A 1S6 Canada; 5 School of Nursing, University of Victoria, Victoria, BC V8P 5C2 Canada

**Keywords:** Suffering, Palliative care, Assisted death, Medical assistance in dying, End of life, Nursing, Qualitative

## Abstract

**Background:**

Intolerable suffering is a common eligibility requirement for persons requesting assisted death, and although suffering has received philosophic attention for millennia, only recently has it been the focus of empirical inquiry. Robust theoretical knowledge about suffering is critically important as modern healthcare provides persons with different options at end-of-life to relieve suffering. The purpose of this paper is to present findings specific to the understanding and application of suffering in the context of MAID from nurses’ perspectives.

**Methods:**

A longitudinal qualitative descriptive study using semi-structured telephone interviews. Inductive analysis was used to construct a thematic account. The study received ethical approval and all participants provided written consent.

**Results:**

Fifty nurses and nurse practitioners from across Canada were interviewed. Participants described the suffering of dying and provided insights into the difficulties of treating existential suffering and the iatrogenic suffering patients experienced from long contact with the healthcare system. They shared perceptions of the suffering that leads to a request for MAID that included the unknown of dying, a desire for predictability, and the loss of dignity. Eliciting the suffering story was an essential part of nursing practice. Knowledge of the story allowed participants to find the balance between believing that suffering is whatever the persons says it is, while making sure that the MAID procedure was for the right person, for the right reason, at the right time. Participants perceived that the MAID process itself caused suffering that resulted from the complexity of decision-making, the chances of being deemed ineligible, and the heighted work of the tasks of dying.

**Conclusions:**

Healthcare providers involved in MAID must be critically reflective about the suffering histories they bring to the clinical encounter, particularly iatrogenic suffering. Further, eliciting the suffering stories of persons requesting MAID requires a high degree of skill; those involved in the assessment process must have the time and competency to do this important role well. The nature of suffering that patients and family encounter as they enter the contemplation, assessment, and provision of MAID requires further research to understand it better and develop best practices.

## Background

The desire to mitigate suffering is the primary motivation of those providing care at end-of-life [1]. However, even when symptoms are well managed, the losses that attend a life-limiting illness, and the realization that one will soon die, can cause suffering [2]. Although suffering has been the substance of philosophic and theological thought for centuries, an empirical focus on suffering only began in the early 1980s [3]. As treatment options at end-of-life become more numerous and sophisticated, understanding the nature of suffering and its role in relation to modern healthcare becomes ever more important. A number of questions form the substantive debates about suffering: What is the nature and normative function of suffering? Who gets to determine when suffering exists? What therapies are acceptable for what types of suffering? What are the moral claims of intolerable suffering? [4]. Bozzaro and Shildmann [4] argued that the answers to these questions are determined by the underlying assumptions about the nature of suffering. They distinguished two typologies of suffering that form these underlying assumptions: a subjective/holistic one informed by the work of Cassell [5] and an objective/gradual one informed by the work of van Hooft [6, 7].

Cassell defined suffering in the following way:

“a specific distress that occurs when an impending destruction of the person is perceived and continues until the threat is done or the integrity of the person can be restored. A person is an embodied purposeful, thinking, feeling, emotional, reflective, relational human individual existing through time in a narrative sense. Generally, all of these parts are consistent and are harmoniously accordant. Suffering, in which all of these parts are affected, variously destroys the coherence, cohesiveness, and consistency of the whole. It is in this sense that the integrity of the person is threatened or destroyed” ([8] p. 436).

An important aspect of Cassell’s understanding was that suffering is holistic, so one cannot tease out aspects of suffering into physical or existential domains. Further, these suffering experiences have a cognitive component; the meaning of suffering is derived from the beliefs and values of the person having the experience [5]. In contrast, van Hooft proposed that persons exist on four levels (biological, appetitive, deliberative, contemplative) and each level has an ultimate purpose toward which individuals strive. Suffering occurs when the pursuit of that telos is disrupted [4, 9]. Unlike Cassell, van Hooft believed that suffering need not contain cognitive content; one need not be aware to suffer. Further, practitioners can intervene to relieve suffering in one of the four domains, which in turn influences the functioning of others.

This distinction between subjective (Cassell) and objective (van Hooft) accounts will broadly determine the extent to which one believes they can clinically assess suffering in an objective sense and the acceptable means by which to treat it. For example, there is the clinical question of whether palliative sedation is warranted in the case of existential suffering or whether it should only be used in the case of refractory physical symptoms [10]. If one holds to Cassell’s view of suffering, this distinction would not be meaningful for it would be impossible to differentiate between the two [8].

Nowhere are these debates about suffering more relevant than in the legalization of, and subsequent use of suffering as eligibility criteria for, medical assistance in dying (MAID): *An Act to Amend the Criminal Code and to Make Related Amendments to Other Acts (Medical Assistance in Dying)* [S.C. 2016, c. 3]14 [11]. Canada’s original MAID law (hereafter referred to as Bill C-14), arose out of the finding of the Supreme Court of Canadian *Carter v Canada* [2015 SCC 5] that the blanket prohibition on MAID contravened section 7 of the *Canadian Charter of Rights and Freedoms:* the right to life, liberty and security of the person and right not to be deprived thereof except in accordance with the principles of fundamental justice [12]. First, the blanket prohibition interfered with the plaintiff’s control over their bodily integrity causing physical and serious psychological suffering, thereby infringing the plaintiff’s right to security of the person.^1^ Second, by interfering with the plaintiff’s “right to make fundamental personal choices free from state interference”^2^ (in this case, “decisions concerning their bodily integrity and medical care”)^3^ the blanket prohibition infringed upon the plaintiff’s right to liberty. Third, by forcing a person in the position of the plaintiffs to make the “cruel choice” between committing suicide “prematurely, often by violent or dangerous means, or… [to] suffer until she dies from natural causes”^4^ the blanket prohibition infringed upon the right to life.^5^ Madam Justice McLachlin, writing for a unanimous court, described the plaintiff’s decision to seek MAID in these circumstances as “rooted in their control over their bodily integrity; it represented both their deeply personal response to serious pain and suffering”^6^; the “constant theme” “running through the evidence of all the witnesses… [is] that they suffer from the knowledge that they lack the ability to bring a peaceful end to their lives at a time and in a manner of their own choosing.”^7^ The infringement of these rights would have been justified by the “principles of fundamental justice” if the blanket prohibition was necessary in order to protect the interests of vulnerable persons. The Court concluded that it was not, citing “social evidence” from jurisdictions allowing MAID and the “preponderance of the evidence from ethicists… that there is no ethical distinction between physician-assisted death and other end-of-life practices whose outcome is highly likely to be death”^8^ In relation to the “vulnerable”, Chief Justice McLachlin observed that “it was feasible for properly qualified and experienced physicians to reliably assess patient competence and voluntariness, and that coercion, undue influence, and ambivalence could all be reliably assessed as part of that process.”^9^

*Carter* concludes with a declaration that those sections of the Criminal Code that created the blanket prohibition on MAID would be declared void *in the following circumstances* (and not void per se): where a competent adult person clearly consents to the termination of life and has a grievous and irremediable medical condition (including illness, disease or disability) that “causes enduring suffering that is intolerable to the individual in the circumstances of his or her condition.”^10^ The *Carter* declaration provides the basis for the subsequent legislation (although the legislation is not, and need not be, identical to it), and the articulation of “intolerable” enduring suffering here is significant, understood in connection with the multi-faceted analysis of suffering in connection with the *Charter* protected right to life, liberty and security of the person that precedes it.

An analysis of the arguments made by interveners in the Court challenge suggested that society was divided in its perceptions of the role and meaning of suffering [13]. According to Beaman and Steele, arguments against the legalization of assisted dying tended to portray religious perspectives of suffering as redemptive, transformative, and a means by which one could sacrifice self to the glorification of God. In contrast, those who argued for assisted dying portrayed suffering as cruel and a threat to human dignity [13].

Such differences in the meaning persons attribute to suffering are apparent throughout the philosophic literature. Aaltola [14] discussed three typologies of the meaning of suffering derived from the works of Levinas, Neitzche and Weil. The work of Levinas was used to typify a negative view of suffering as “alien to humanity,” a “destructive, disabling, de-subjectifying, and de-humanizing force” (p. 24) that must be avoided and eradicated at all costs. This eradication “re-establishes our humanity and morality.” (p. 25) In contrast, an ennobling view of suffering, as characterized in the works of Neitzche, portrays suffering as developing capacities that cultivate our minds and, hence, make us more fit for the world. Finally, the work of Weil was used to typify a transformative perspective of suffering in which the illusions of “self-creation and independent subjectivity” (p. 30) are traded for a more connected and authentic view of the world. “Suffering emerges as useful, but not heroic.” (p. 30) Weil’s view of suffering provides a bridge between the dehumanizing perspective of Levinas and the heroic perspective of Neitzche. The decision made by the Supreme Court that ultimately led to the legalization of MAID reflected a perspective of suffering as primarily dehumanizing and something to be avoided, according to Beaman and Steele. “The law as it stood was characterized as imposing additional and unnecessary suffering on those who were terminally ill. In this view, suffering was not something with redeeming spiritual qualities, but rather something to be treated and avoided.” ([13] p. 12).

In March 2021, MAID was extended in Canada to those whose natural death is not reasonably foreseeable under *An Act to Amend the Criminal Code (Medical Assistance in Dying)* SC 2021, c. 2 (hereafter referred to as Bill C-7). That legislation set out two distinct processes for accessing MAID, one for those whose natural death was “reasonably foreseeable” and one for those whose natural death was not “reasonably foreseeable.” In relation to the latter, assessors and providers of MAID are required to:

“ensure that the person has been informed of the means available to relieve their suffering, including, where appropriate, counselling services, mental health and disability support services and palliative care and has been offered consultations with relevant professionals who provide those services and that care;

ensure that they and the medical practitioner or nurse practitioner have discussed with the person the reasonably and available means to relieve the person’s suffering and they and the medical practitioner or nurse practitioner agree with the persons that the person has given serious consideration to those means.” [15, 16]

For the population whose death is not reasonably foreseeable, there is a greater responsibility on the part of assessors and providers of MAID to ensure that persons have given serious consideration to options available to relieve their suffering. In contrast, no such responsibility is implied for those whose natural death is reasonably foreseeable.

The first annual report on MAID in Canada, authored by Health Canada prior to the 2021 legislation, made an intriguing statement about the role of the practitioner in assessing suffering.

It is not the practitioner’s interpretation of the intolerability of an individual’s suffering; only the individual requesting MAID can determine whether their suffering is unbearable. That being said, practitioners must not provide MAID if they do not feel that the patient meets the eligibility criteria. ([17] p. 31).

Such statements are difficult to interpret when suffering is to be considered as context. Does this mean that there is room for clinical judgement about intolerable suffering or that clinician judgement should only be applied to the remaining eligibility criteria? The report outlined the causes of suffering that led to MAID. Of the 7384 deaths reported in 2020, the most common were “loss of ability to engage in meaningful life activities” (82.1%) and loss of ability to perform activities of daily living” (78.1%) (p. 32). Inadequate control of symptoms (or concern about it) were reported in 56.4% of cases [17]. It is important to note that this descriptive category of suffering included those with anticipatory concerns about inadequate symptom control, not just those who were currently experiencing inadequate symptom control.

The emphasis on suffering in the MAID legislation, and the clinicians’ responsibility to satisfy themselves that the means to relieve suffering have been addressed, places assessors and providers squarely within the contentious conceptual landscape of suffering. Is suffering an experience that can be objectively determined and thus amenable to diagnosis and treatment? Or is it a subjective experience in which diagnosis and treatment could be considered an affront to the existential human experience? Is there room for narratives of heroism and transformation or is suffering simply something to be extinguished as an afront to human dignity? Such philosophic questions confront MAID practitioners on a daily basis, and yet, we know little about their experiences of disentangling such conceptually thorny issues. Therefore, the purpose of this paper is to describe nurse and nurse practitioner’s experiences of encountering and making sense of suffering in the context of MAID. In Canada, registered nurses provide continuous care to patients throughout the MAID process and are often in coordinating and oversight roles for MAID programs and teams. Most provinces and territories in Canada use interdisciplinary teams and/or central coordination services to provide MAID within a designated geographic region. Primary care providers may choose to be involved in the process but are not required to do so. Nurse Practitioners are legally permitted to independently assess patients for MAID eligibility and provide MAID to eligible persons. As such, registered nurses and nurse practitioners provide an important angle of vision into MAID care in Canada.

## Methods

The aim of the larger study within which this analysis is situated is to describe the longitudinal development of MAID and palliative care in the Canadian context (2017–2024). In this paper, we present findings specific to the understandings and application of suffering in the context of MAID from nurses’ perspectives.

Design: The design is a longitudinal qualitative study using semi-structured interviews and document review as data collection strategies. Data collection is occurring at three time points: 2018/2019, 2020/2021, 2022/2023. With each wave of data collection, previous research participants are contacted and new participants are recruited. Data was collected during the fall of 2020 and spring of 2021, a time when Bill C-7 was under review and early implementation. The sample (*N* = 50) from which this data was derived consisted of 34 nurses and 16 nurse practitioners, 26 of whom had been interviewed previously and 24 of whom were new participants interviewed in 2020/2021. The mean number of years of experience was 17.9 (SD 11.8) (see Table 1 for demographic characteristics). This study underwent ethical review by the behavioural research ethics board at the University of British Columbia.

**Table 1:** Participant characteristics (N = 50)

Characteristics	Number	Percent
Nurse Designation		
Registered Nurse	34	68
Nurse Practitioner	16	32
Ethnicity		
Caucasian	45	90
Other	5	10
Province of Work		
BC	23	46
Ontario	21	42
Other	6	12
Geographic Context^a^		
Urban	41	82
Rural	28	56
Remote	8	16
Population worked with^a^		
Specialized palliative	26	52
Primary care	17	34
Medical surgical	13	26
Other	11	22
Number of MAID deaths experienced		
0–9	15	30
10–24	17	34
25 or more	18	36

^a^Categorical responses not mutually exclusive

Sample: New participants were recruited using purposive and snowball sampling. Recruitment bulletins were distributed through national nursing agencies and through the Canadian Association of MAID Assessors and Providers. Strategies included email, Facebook, Twitter and word of mouth. We recruited from every English-speaking province; however, we did not actively recruit from the three Territories in Canada as MAID was an infrequent occurrence in these areas. Previous research participants were contacted through email.

Data Collection: All participants signed an informed consent and then took part in a semi-structured telephone interview. Telephone interviews were necessary to access a pan-Canadian sample. Interview questions probed around nurses’ understandings of suffering in the context of MAID and strategies used to better understand the suffering narratives of clients. Some participants were nurse practitioners responsible for acting as MAID assessors and providers; other participants were registered nurses in roles in which they were responsible to provide care to those undergoing the MAID process. As a result, we were able to obtain a range of experiences. Interviews were audio-recorded, transcribed verbatim, checked for accuracy, and downloaded in NVivo (QSR) software for analysis.

Data Analysis: Inductive coding followed the principles outlined in Interpretive Description [18]. Coding was started after a number of interviews had been completed so that the investigators were familiar with a large portion of data. Two investigators read the data in its entirety and co-constructed open codes which were then refined using analytic insights from three additional investigators. These open codes were used to code all remaining transcripts. Constant comparative analysis was used to compare and contrast data to identify patterns and commonalities [19]; codes were further refined through this process. Themes were then developed and a narrative account was constructed to answer the research question. This narrative account was further developed and refined with input from all team members. Additional analytic insights were captured using memos and field notes; however, these were not coded as part of the data set.

## Results

The findings conceptualize nurses’ experiences with suffering in the context of MAID. We provide an in-depth account of the suffering that leads to a request for MAID. We describe how nurses learn to hear the suffering story to clarify that this is the right choice for the right person at the right time. Though MAID is a treatment designed to end suffering, the process itself can result in a different and quite distinctive form of suffering. This occurs from having to decide whether having MAID is the right choice for all involved, from undergoing the eligibility process with the risk of being deemed ineligible, or from experiencing the heightened work of dying in preparing oneself for MAID. However, to better understand these suffering experiences, it is important to provide context around the suffering of dying from nurses’ experiences and so it is there that we begin (See Table 2).

**Table 2:** Thematic findings

Theme	Sub-themes
The Suffering of Dying	Work and loss
	“Treating” existential suffering
	Institutionalized suffering
The Suffering that Leads to MAID	Un(known) dying
	Un(predictable) dying
	Un(dignified) dying
Suffering as Eligibility?	Hearing the suffering story
	Suffering is what the person says it is
	Right person, right time, right reason
The Distinct Suffering of MAID	Doing the right thing
	(In)eligibility
	Heightened work of dying

### The suffering of dying

Understanding the experiences of dying, particularly within our modern healthcare system, is important to an appreciation of the suffering that leads to the desire for an assisted death. Here we describe how participants characterized suffering as work and loss, how they grappled with treating existential suffering and the system issues they felt that contributed to a form of institutionalized suffering.

#### Work and loss

For these participants, the suffering of dying was characterized as work and loss. “*Suffering is work because it tests us. Right? It tests our boundaries, our tolerance, it pushes on us, it pushes on our acceptance of the edges of where we would or wouldn’t accept a loss of self.”* (P64) This labour entailed learning to accept the loss of the multitude of abilities that make up the sense of self, and hence, make life meaningful. “*I would frame it (death) as loss, the loss that just keeps on losing. One thing and the other thing, the next thing, the next thing, the next thing until people lose the desperation about not being here anymore. They accept that there is nothing more to be here for.” (*P40)

This labour of dying entailed continually weighing the advantages and disadvantages of being in the present condition. Participants further described this suffering labour as needing to reconcile the knowledge that ones’ life is coming to a close with the desperate hope of wanting to live. “*What I describe is the journey from head to heart. People are facing the end of their life so they have to go from the head to the heart and back again where they’re figuring out what to do with the cognitive processing and the feelings that arrive out of facing the fact that I’m going to die.”* (P64)

#### “Treating” existential suffering

When describing the suffering of dying, study participants often differentiated between a suffering associated with physical symptoms and an existential suffering. In most cases, participants believed that high quality palliative care could relieve the suffering associated with physical symptoms; however, existential suffering was a different matter. As one depicted it: “*That existential suffering, that grief suffering, the anticipatory death suffering, the weight of leaving your family behind suffering, all that sort of stuff.”* (P11)

Participants differed in their opinions about the effectiveness of palliative care in treating this existential suffering; even those with extensive palliative experience spoke to the refractory nature of this suffering. Some told stories of working successfully with patients using meaning-making activities; others explained the difficulties of working with patients who held to patterns of thinking that contributed to existential despair.

*People are very complicated and sometimes we, being health care providers and spiritual folks, just can’t relieve existential suffering all the time. And if somebody is feeling like their whole life is so black and that they’ve got pain and that they can’t have relief and that they can’t resolve whatever it is that needs to be resolved, then I think in a very small percentage of cases, we can’t (relieve that suffering), for whatever reason.* (P98)

However, speaking of this suffering as refractory assumed that such suffering should be the focus of palliative treatment. In fact, for many participants the suffering required one simply to be present and bear witness. *“The hardest thing that we have to do is bear witness to that suffering and stand by and support and offer opportunities for self-exploration and self-expression and figuring out how to support the person to work through that angst.”* (P64)

#### Institutionalized suffering

Although the suffering associated with physical symptoms was seen to be potentially amenable to treatment, participants suggested there were complicating factors such as patients’ experiences with the healthcare system and lack of timely access to palliative care. Study participants who had worked as nurses in the acute care system for many years reflected on how much patients had suffered from their treatment. Specifically, many patients had a long history of accepting treatments that negatively influenced their quality of life in the hope of a longer life. However, this acceptance of the burden of treatment changed over time. The following participant described this treatment-induced suffering in relation to the knowledge that it could be ended quickly through MAID.

*If I told you every day, I’m going to come and butt out a cigarette on your arm. Every single day, I’m going to come, butt out that cigarette, there’s nothing you can do about it and I’m going to do it at times when it’s inconvenient and sometimes I’m going to do it when you’re having a great time with your kids and sometimes I’m going to do it when you’re sleeping but I’m going to come in every day and butt out a cigarette on your arm. And then, one day you say to me, 'In two weeks from now, I’m going to die. You can keep butting out that cigarette but I know for the next two weeks, there’s only so much more you can do to me.'* (P70)

Patients’ ongoing relationship with treatment-induced suffering resulted in “institutionalizing suffering” (P70) in such a way that persons could be unwilling to seek relief from a palliative team that was affiliated with the same system that they had struggled with for so long. This institutionalized suffering was further compounded when patients felt they were not well-informed about their treatment choices and ended up worse after treatment than before. Even with high quality palliative care, participants recognized that symptom relief and treatment constituted a series of trade-offs in relation to quality of life. “*We give them a drug that’s supposed to get rid of the pain but gives them awful, scary hallucinations and they’re screaming at night. Like, who wants to live like that?”* (P48)

Further complicating the ability to adequately address physical suffering was the lack of resources to meet patients' needs at end of life. Participants spoke of persons who had no social support, were poorly housed, were living in poverty, or had family caregivers who were over-burdened. They described palliative systems in which it was impossible to provide timely and responsive treatment for symptoms. “*I had one lady in a pain crisis in the middle of the night who had to wait three and a half hours for the closest on-call nurse.”* (P82) These challenges in care were perceived to be particularly detrimental because they were layered onto the sufferings of persons who were already coping with the work of dying. It was in the context of this dying work that participants understood the suffering that could lead to the request for an assisted death.

### The suffering that leads to MAID

When asked about the types of sufferings that lead to a request for an assisted death, participants described several common situations: the unknown of dying; a desire for predictability, and the loss of dignity.

#### Un(known) dying

In our current societal context, few individuals have first-hand experience with death; death is the great unknown. *“It’s just the unknown. Just not knowing what to expect.” (P1)* Instead, perceptions of suffering in dying are formed by stories, media images, imagination, and in some cases life experience in close proximity to death. This unknowing can lead to fear about the process of dying. “*I think that the fear of that suffering, coupled with, really truthfully, lack of education, lack of expertise in the area of end-of-life care [is what forms opinions]. But truthfully, in probably 500 patients minimum I’ve sat with, very few really suffered at the end of their life.”* (P47)

One participant described the influential effect of the media on perceptions of how dying should ideally occur. “*You give them something and they get comfortable and then they die…they’re cognizant to the last breath and then say, ‘I love you’ or something really meaningful and then drift off*.” (P78) Such media stereotypes were more in keeping with the process of a MAID death than with the often long and arduous process of a natural death.

However, not all perceptions about death were ill-informed. Participants described how some persons requesting MAID had provided care for a family member through a protracted dying process. Those experiences made them determined to not take the same path. One participant described an elderly MAID candidate who had been so traumatized by caring for her husband over a long illness course that she was eagerly awaiting her MAID provision day. “*I think she had huge suffering. She was just so fearful of becoming ill that she just couldn’t wait to get to that ten-day mark*.” (P67)

#### Un(predictable) dying

A MAID death provided a sense of predictability amidst the unpredictable process of dying. It is important to note that participants did not necessarily equate this need for predictability with fear, but rather equated it with a desire to maintain ownership over oneself until the end. This approach to death was often apparent in the ways that persons had preferred to conduct their lives in general. “*People who want to assert themselves and have control over their lives. They’re used to figuring things out for themselves*.” (P5) This predictability was particularly important when their “*body had lost control.*” (P72) Participants further explained how this need for predictability was not always linked to the alleviation of physical suffering. For example, when exploring a MAID applicant’s suffering, one participant asked if they would want to live longer if they could get more good days than bad days. The person responded: “*The trouble is I can’t choose when I am going to have a good day or a bad day. And that in itself is suffering*.” (P40) Participants spoke of the false stereotype of the person desiring MAID as one with a “*furrowed brow*” and “*on their deathbed*.” (P14) Instead, one participant told the story of a young woman who chose to walk out to her balcony to sign her consent for MAID there, because it symbolized her ongoing personhood and autonomy.

This predictable approach was also intensely pragmatic. This was evident when participants likened this approach to MAID to their sense of the philosophical attitudes characteristic of those who live close to the land. For example, several participants described the pragmatic independence characteristic of those who had grown up in rural and remote locations. “*Life is dirty fingers and being in touch with the earth. And the earth gives forth and takes away. The best visual I can think of is dirt under your fingernails*.” (P95) This participant likened MAID to a farmer’s approach to animal husbandry that cherished life while also dealing pragmatically with death (e.g., we should probably euthanize the horse before winter so we can give him a good burial). Others described the pragmatic decisions that some persons took to receive MAID in their rural communities because there were no adequate palliative supports and they wanted to die at home. Such considerations of predictability and pragmatism were less about control and more about an approach to life in general, and a certain quality of life in particular.

#### Un(dignified) dying

Participants frequently used the idea of a line to describe transition points where persons believed their dignity was compromised to the point where they no longer wanted to live. This was the proverbial *“line in the sand”* (P84) that, when crossed, required action. This line was often crossed when persons could no longer manage their own bowels or do self-care. “*Then, she crossed a line of her own making, a line of ‘this is intolerable now’. And it was regarding incontinence. So, she then asked her family for the chance to explore MAID. In requesting MAID, she was in a sense saying I am suffering now.”* (P5)

The line was different for everyone, but if crossed, might well meet the criteria for intolerable suffering as individuals saw themselves as “*no longer living but only existing.*” (P99) This idea that one could exist but not live was described by another participant as having elements of our lives dying at different times. *“I’m so pleased that we have MAID because you have people that they are ready to die before their body is ready to let go. Like, dying happens on all spheres. Right? Spiritually, socially, emotionally, physically.”(P76)* The idea that death could happen to a person on different levels and at different times, and that it was up to the person to decide when that line was crossed, had implications for using suffering as an eligibility criterion for MAID.

### Suffering as eligibility?

The various reasons for requesting MAID described above point to the challenges of incorporating suffering into the eligibility criteria for MAID. Perceptions of how death should go, or the need to keep ownership over one’s body, call into question our traditional ideas of suffering as the “*furrowed brow*” (P14) in which suffering is evident to the outside observer. Here we describe how nurses heard, framed, and weighed suffering in the context of a MAID request.

#### Hearing the suffering story

Nurse participants who acted as MAID assessors told rich stories of trying to understand the suffering that motivated a MAID application. They began from an assumption that not every request for MAID was actually a request to die; rather, it was seen as an invitation to a conversation about the experiences of a person’s life and illness. “*I’ve had so many conversations with people about wanting to pursue MAID and then when you have a conversation and you realize they don’t want to pursue it at all. It’s other things that they’re crying out for*.” (P84) This invitation to conversation required a fulsome exploration of the details and conditions in which applicants found themselves. “*Suffering is one of those catchall phrases that means different things to different people and I think we can do our best to climb into the heads and the hearts of the people describing it and just understand it.”* (P40) Participants described an array of approaches and questions they used to explore the suffering experience in as much depth as possible, questions that were designed to get around some of the hesitancy that applicants might have in sharing their story. *“‘We’re Canadians and we don’t complain. Don’t be Canadian for a minute and complain. Tell me. Tell me what is the hardest part about this and don’t hold anything back.’ And just listen to them tell their story and again. I don’t listen to it with a MAID focus or a palliative care focus, I listen with patient-centred focus*.” (P93) However, getting into this personal world was not easy, and it required time to gain confidence in this ability. “*I’m a nurse. I’d rather talk about your constipation than your relationships and your emotional pain because it’s just more tangible.”* (P67) She went on to explain that there was an art to entering this kind of conversation work, and it was easy to doubt your expertise at managing it appropriately.

#### Suffering is what the person says it is

The goal of this type of conversation that nurses had with their patients was to know the person in as much detail as possible in the limited time available, not to determine whether someone was eligible for MAID based upon their suffering. In fact, the legislation as written had explicitly prohibited “second guessing” the patient with respect to that aspect. “*That’s been really hard to get into peoples’ heads that suffering is the one eligibility criteria of the assisted dying clause that says that the MAID providers don’t assess whether it’s true or not.”* (P86) However, it could be difficult for experienced assessors to communicate this message to those less knowledgeable about the legislation. When clients appeared otherwise well, and had no visible indicators of suffering, it could be challenging to accept that there was a limited role for clinical judgement about that suffering. “*The Criminal Code was quite clear that suffering is what the patients deems unacceptable and so we struggle with people saying, ‘Well, that person’s fine. They’re still up and walking.”* (P4)

#### Right person, right time, right reason

This person-centered exploration was required to ensure that this was a right choice for this person at this time. Hearing the suffering story assisted all concerned in knowing whether the options to relieve suffering were known by the applicant and had been tried. “*Well, if you think that that’s intolerable, what if I said to you that I could take that away? Would that be as intolerable? And in nine times out of 10, I have found people say ‘No. I didn’t realize that that could happen*.’ *You can’t expect the world to be educated in all these things.”* (P82) It also enabled them to determine whether applicants were seeking MAID because of fears that might be unfounded. One participant described working with a long-term client who was terribly afraid of death and it was this fear that prompted her to request an assessment for MAID. She described having a conversation about all the things the client was still enjoying in life and ultimately proposing that it was perhaps not yet the right time because she was “*not suffering enough.”* (P94) However, it is important to note that statements such as these were always accompanied by reassurances that clients would be helped when the right time came.

Even as assessors relied upon applicants to judge what constituted intolerable suffering for them, applicants were not always sure what that meant. This participant described the typical questions that applicants asked themselves.

*‘Well, is my suffering intolerable? I'm not suffering as much as, you know, other people in X, Y, and Z situation. Maybe my suffering is tolerable. Am I being weak because I'm, you know, accessing MAID?’ From a legal perspective, I think it's good that it's from the patient perspective but it does put a little bit of a burden on the individual to figure out what is their intolerable suffering and what does it mean to them?* (P81)

In some instances, applicants for MAID were so concerned that they would be deemed ineligible that they felt they had to demonstrate the depth of their suffering to the assessor. Experienced assessors described how they had learned to tell persons up front that, although they were there to hear their story, the law did not require them to make a determination about whether or not they were suffering.^11^

### The distinct suffering of MAID

The decision to pursue MAID could provide considerable relief from the otherwise unrelentless work of illness and dying. But, the process itself could result in new forms of suffering. Deciding whether MAID was the right thing to do, taking the risk of being deemed ineligible, and undergoing a heightened death experience were experiences that brought forward distinctive dimensions of suffering.

#### Doing the right thing

Making the decision to pursue a MAID death, or even apply for an eligibility assessment, was not an easy one. Although applicants typically knew what they wanted, the decision was more difficult once they considered the reactions of family and friends. Families were an important part of the dying journey and so suffered vicariously whether the patient chose an assisted death or not. *“The suffering that comes with all the losses not only affect the patient but their family…the suffering that they’re experiencing with their losses and that’s all one piece.”* (P47) However, a MAID death was different because it meant that the individual was making an active choice to die early rather than passively awaiting ‘natural’ death. One participant described this process of decision-making as a drama in which all of the actors played an important part. “*When having to reckon with choosing MAID, each of the people in this drama, the sons, the daughters, the wives, the husbands, have to get to that place too, so it is a tortuous journey. It’s not straightforward, or if it is, people are very, very lucky*.” (P14) Playing out this drama could cause suffering for those who disagreed with the choice of MAID, suffering that required sacrifice and courage.

*I think the only people that suffer are the family or loved ones that don't feel comfortable with the idea of MAID. They have that battle in their own minds and they have this loved one that they want to help. I think it's really hard on them, super hard on them. And you can see the emotion and the strain that they go through. They may not believe in it themselves, or want that, but they will do it because they know that's what their loved one wants. It's just amazing to see this sacrifice on their part and they just dig in there and do what they need to do for their loved one.* (P48)

Family members wrestled with the idea that MAID was suicide, with grief over potential time lost, and with the fear that the souls of those involved in MAID would be in jeopardy. The perception that MAID is a form of suicide, and in some cases the consequent fear that their loved one would suffer in the afterlife, was a real and present concern.

*Some of the trickiest ethical cases I've been involved with is when family members feel quite strongly that their loved one should not proceed with MAID, that they are committing suicide and because of that they will be going to hell and then they won't be able to see their loved one in the next life. That can cause a lot of moral distress for the family but also for the patient themselves at end-of-life.* (P81)

This participant went on to stress how the possibility of permanent separation could actually exacerbate the psychological and existential suffering that MAID was intended to alleviate.

Other family members ruminated on the amount of time that was lost if MAID was chosen. “*I get a lot of questions such as ‘how long would it have been for her to die naturally if we didn’t do this? Would she have been gone in a week? 10 days? tomorrow?’”* (P14) This need to know the time until a natural death would occur could indicate a sadness over time lost together or a need to weigh the moral implications of the choice. If the number of days allowed to a person are ordained (e.g., in the case of religious perspectives in which God has pre-ordained the length of each person’s life), then presumably a MAID death closer to a natural death would be the least morally culpable option.

Other participants mentioned the significant impending role change of family caregivers caused by a MAID death. “*Your life is looking after your mother…And that’s your purpose and then your mother doesn’t want you to do that anymore*.” (P14) The choice of MAID could also hold the unspoken implication that the family caregiving had been insufficient. Overall, the choice to pursue MAID was a far-reaching one that required significant emotional and moral work on the part of everyone involved. Friends and family involved in this decision typically each ended up in one of three situations: fully supportive of the MAID choice, unreconciled but supportive of choice anyway, and unreconciled and unsupportive. (P14)

#### (In)eligibility

Once persons had taken the step of requesting a MAID assessment, the outcome of the assessment could either relieve or exacerbate the suffering that led to the request in the first place. Participants described the sense of relief that patients experienced once they were found to be eligible. “*A peace comes over patients when they have been approved for MAID. And even if they’re not going to choose it, they feel a comfort and a sense of ‘I don’t have to go through these horrendous final days.’”* (P72) However, participants used strong language to describe the suffering that occurred if applicants for MAID were found to be ineligible.

*There's massive suffering for them. They feel let down by the system. They feel let down by their MAID provider. They feel let down by the law and they don't understand why they're not considered eligible*. *It's really hard for them to reconcile in their head that people could have different opinions about this when they're living it every day and they're suffering. And they don't understand. (*P86)

The impact was perceived to be so extreme because persons who applied were suffering greatly, did not enter into the decision lightly, and once they had made the decision, gained hope that was subsequently dashed when they realized this was not an option for them. Participants shared how, after some early dramatic experiences of what could occur after someone was deemed ineligible, they had learned to provide additional support for this population. “*It is devastating and those are the patients who need the most support. And we do talk about ineligibility as part of our initial contact so that people understand not everybody’s eligible and we’re going to support them either way*.” (P92)

#### Heightened work of dying

Once eligibility for an assisted death had been granted, applicants still had a number of decisions to make and preparatory work to do. Now that they were in control of the process, decisions that might have otherwise been left to chance in a natural death now had to be made, such as the timing, place, and rituals of death and who should be present. Waiting for that final day and hour could be intense as the emotional work of dying now had a time limit. Finally, the final moments of death were intense, leading some family to feel like they were actors in a stage performance.

Although successful MAID applicants were not required to set a date once they had been deemed eligible, some felt a need to do so. “*As soon as they start the paperwork, the clock is ticking and they have to set a date and that’s really anxiety-provoking for them.” (P48)* However, unless persons were at risk of losing cognitive capacity to consent, they were required to fulfill the legislated 10-day waiting period. The emotional work during this time could be intense. Persons had to decide who should be present, who should be told, and the practicalities of planning the MAID death. Family dynamics that pre-existed this decision were often accentuated, and in some cases, the patient’s choice was used to leverage positions associated with long standing tensions. “*So, that sort of emotional family dynamic, a life long-lived and now to be ended, I think that causes more duress in people than we give it credit for and I don’t know how to fix it. I don’t know how to make it easier.”* (P40) This emotional work was exceptionally tiring for family caregivers who were often at the end of a long caregiving commitment. “*The spouse was bracing herself for this last big event. She was already so tired and she was private, they had done things privately. But now there were friends and a big gathering. This was one more thing she had to get through.”* (P67)

The final moments of waiting for the team to arrive to provide the MAID were particularly acute. This nurse, who had arrived early on one occasion to ensure that everything was ready, shared her experience of that time. “*He just kept saying, ‘when is the doctor coming? I want this to be over.’ I kept telling him 20 minutes but it was too long. I didn’t know what to do*.” (P37) The heightened intensity of a MAID death was explained further by this participant. She spoke of how, in a natural death, persons often chose to die when their loved ones leave the room. There is some comfort in that uncertainty of time because one never knows when they have to face the final moment together. However, a MAID death is an event in which others are often invited to take part. “*In a MAID death, time stands still. Everyone knows the moment feels like they are onstage and what is normally dissolved in a sense of uncertainty must now be confronted with certainty. There is no diffusion of the pain or the anxiety.”* (P67) Despite this heightened experience throughout the time of preparation and death, once the procedure was over there was often a sense of profound relief. *“Most times I see a sense of relief that the family have made it through. Like, it’s this big buildup.”* (P68)

Thus, suffering played a powerful role in the decision to seek MAID, in the discussions around eligibility for it, and in the experiences of patients and families in the enactment of this new kind of end-of-life option. Nurses working with these patients, regardless of their role as assessor, provider, or general supportive caregiver, were very much caught up in bearing witness, guiding and supporting suffering experiences.

## Discussion

MAID is one of the few areas of practice carried out by health professionals that is directly regulated by legislation. As a public act in a democratic society, the ideas of who should be eligible and the required safeguards have generated robust debate from a variety of stakeholders with varying degrees of experience with suffering. In turn, society has delegated this task of assessing for and providing MAID to healthcare providers who serve as the gatekeepers. As such, it is vital to understand how healthcare providers involved in the MAID process, from initial consideration to provision, understand and evaluate the suffering of those considering this option. Clinicians’ acquaintance with, and views of, suffering will influence how they view and treat client suffering [20]. Findings from this study provide important insights into nurses’ understandings of suffering in the context of MAID, the ways in which they learn about that suffering, and the different types of suffering that characterize the work of a MAID death.

### Beyond the furrowed brow: intimate understandings of the nature of suffering

An important part of the ongoing conversation about the role of suffering in assisted death are the suffering histories that clinicians bring to their client encounters [21, 22]. In this study, nurses demonstrated a profound empathy for two origins of suffering to which their clients were at risk, the suffering of living with a progressive illness and the suffering induced as a result of the treatment of that illness. There is a robust body of literature providing an overview of suffering at end of life [2]. However, only recently have we begun to provide evidence of the suffering trajectories of those who are travelling the chronic illness trajectory toward palliative care [23, 24]. This transition between chronic illness and palliative care is characterized by increasing symptoms, intensive treatment, and diminishing quality of life. Patients hover in a liminal state between life and death, a phenomenon that has been referred to as ambiguous dying [25, 26]. Suffering during this time is a result of both symptoms and treatment burden that includes physical, financial, temporal, and psychosocial demands. The degree of burden is largely influenced by family support and the quality of relationships between healthcare practitioners and patients [27]. When this patient/provider relationship is characterized by a lack of attention, understanding, or communication about patient's needs, iatrogenic suffering develops [28]. Therefore, in this study nurses recognized that the suffering they were encountering in clients requesting MAID was multifactorial and often influenced by the very system that had tried to heal them.

Nurses’ understandings of suffering were also evident in how they described the reasons clients might choose MAID, understandings that were more nuanced than what is available in public reports [17]. They understood how housing, poverty, and social support might influence the decision for MAID. They understood that patient decisions could be based upon a lack of knowledge or stereotypes of the dying process. They understood that predictability and pragmatism played important roles in the choice of an assisted death. And most importantly, they understood that there was a personal line, that when crossed, meant that one no longer felt alive, or valued living over dying. This knowledge was so well-developed because, rather than being objective observers, these nurses had born witness to such suffering over long and varied careers. The nature and consequences of such bearing witness to suffering, particularly if that suffering is determined to be senseless, have been well documented [29].

Such intimate knowledge inevitably raises the question of what impact patients’

suffering experiences might have on the relational process of working with clients who are considering MAID. Studies have suggested that one of the greatest sources of nurses’ moral distress is overtreatment at end of life [30]. Could this create a sense of over-identification with patient suffering that in turn influences their perceptions of MAID as a preferred treatment option? Early studies of nurses’ experiences with MAID in Canada suggested that these “beautiful deaths” were actually “transforming” nurses’ experiences of suffering [21]. In light of such transformational experiences, one cannot help but wonder if nurses’ biases might be weighted toward a MAID death with subsequent implications for their assessments. In a Dutch study that explored physicians’ application of the unbearable suffering criteria, participants described how they had to balance patient descriptions of suffering with their own perceptions of how they would feel in that situation and their own values and beliefs about what constituted a suffering that could justify euthanasia. As such, these physicians were highly reflective about how their own experiences and values entered into the assessment process [31]. Nurses in this study demonstrated a similarly reflective process.

### Climbing into heads and hearts: the subjective objective dance

An important finding from this paper was the ways in which nurses sought to better understand the suffering stories of their patients, what one participant described as “climbing into heads and hearts.” They described strategies they used to encourage clients to overcome their reluctance to complain or to minimize their suffering and the art of balancing a complete acceptance of the suffering story with the need to determine whether this choice of MAID was for the right person, the right reason, and the right time. Examples of this included the participant who told her clients to complain and not hold anything back or the participant who explored the changes that would be needed for the client’s suffering to become tolerable.

Such an approach reveals both Cassell’s [8] understanding of suffering as holistic and van Hooft’s [6, 7] understanding as suffering as dimensional. It was the interplay of these understandings that allowed nurses to accept the suffering of their patients while still exploring avenues through which to ameliorate that suffering. For example, as nurses took the time to hear patients’ historical stories of suffering, they were acknowledging Cassell’s emphasis on “the individual existing through time in a narrative sense” ([8] p. 436). Hearing these narratives helped the nurse to determine whether the desire for MAID was something that was in keeping with who this person was, not just in the moment of assessment, but over time. In doing so, nurses felt it important to ensure that this was an authentic choice for this individual.

Ahlzen [32] reflected on the relationships between suffering, authenticity, and an assisted death. Authenticity is reflected in our ability to be at home in bodies that enable us to realize our unique values, wishes and beliefs. Once we no longer feel at home in our bodies then our sense of authenticity is lost. Thus, Ahlzen argued, the choice to hasten death is just as much about authenticity as it is about autonomy and dignity. However, in making this observation Ahlzen also argued that, if that is the case, it is important for healthcare providers to have enough knowledge of the person to understand what constitutes authenticity for that person, and that this is only possible when healthcare providers have long-term relationships with clients. When MAID is delivered through healthcare providers who do not have long-term relationships with clients, such as we find in the context of Canadian MAID teams, establishing authenticity may be difficult and rest primarily on the patient’s performance of credibility. Although a number of nurses in this study were not considered the “most responsible provider” for their clients, they recognized the importance of taking the time to establish the authenticity of the request and its occurrence within a congruent life story. Such establishing of authenticity has been widely reported in the literature relating to patients’ wish for a hastened death [33, 34].

Yet, even as nurses gleaned the story of suffering, they sought ways to tease out its origins and address aspects of that suffering. This was evident in the data when nurses asked questions about what might need to change for a patient to no longer desire an assisted death or when they sought to educate clients about what was possible for alleviating their suffering. In doing so, nurses were reconciling the tensions within the MAID legislation by refusing to place judgement on the suffering story while satisfying themselves that the suffering was not as a result of misinformation or lack of attention to amelioration strategies.

### Ongoing moral claims: the suffering of MAID

MAID is the means to alleviate suffering in the public discourse; however, little has been published about the ways in which MAID can cause suffering. Nurses in this study described a developing understanding of the suffering that arose from the moral wrestling of determining whether this was a good choice, the suffering of being deemed ineligible for MAID, and the suffering from heightened death work.

The decision to pursue MAID is often viewed as an important aspect of autonomy and control over one’s life [35]. However, nurses in this study recognized that few patients made this decision independently without considering the multiple effects on family and friends. The evidence on the effects of MAID on family is compelling. Two recent reviews [36, 37] described the range of conflicts, burdens, and cognitive and emotional work required of families through the process of preparation for MAID. Similar to the findings of this study, healthcare providers working within a context of MAID consistently reported that working with families is one of the most challenging aspects [38, 39].

Nurses in this study tried to avoid having a patient deemed ineligible for MAID, even to the point of pre-screening assessors to ensure that their understandings were congruent with the law. In doing so, they recognized that for many persons, MAID was less valuable as a treatment option than as a fall-back plan in case their suffering became too intense. Nurses recognized the irony that, from patients’ perspectives, the primary reason to seek MAID was suffering, and if suffering was patient-defined under the law, then a finding of ineligibility was simply one more experience of a system that induced suffering. As such, nurses felt a profound moral responsibility to ensure that patients did not find themselves in that situation. This deep sense of personal and professional responsibility is evident throughout the literature describing nurses’ role in relation to assisted death [40].

Finally, nurses recognized that even though the MAID death was typically peaceful, the waiting period before the procedure could produce heightened suffering. At times, they felt helpless to intervene in the face of such suffering. However, this does explain why past studies of nurses’ experiences with MAID suggest that nurses tend to characterize this as a brave choice and why they feel such intense pressure to get the last moments just right for patients and families [38, 41, 42].

### Limitations

Study limitations should be considered when interpreting these findings. Data was collected from nurses’ perspectives and so ideas of what patients and families experience are filtered through their perceptions. Interviewees were largely proponents of MAID and so this provides one angle of understanding, which could look quite differently if viewed from a conscientiously objecting standpoint. This sample of nurses had many years of experience within the healthcare system in a variety of roles, many of these within critical care and high acuity areas. These experiences would profoundly shape their perceptions of concepts such as iatrogenic suffering.

### Clinical implications

Findings from this study have important clinical implications. First, they point to the essential knowledge and skills that healthcare providers should have to engage in conversations with those considering MAID. Healthcare providers need to have phenomenological knowledge about what it is like to travel the dying trajectory within healthcare systems and clinical communication skills to elicit the suffering story. Only then will the moral and legal obligations of accepting patients’ suffering stories and attempting to ameliorate that suffering be fulfilled. Second, these conversations take time. Time may be less of a factor if healthcare providers have longstanding relationships with clients, but in the Canadian system where MAID is often delivered through specialized teams, this is not always the case. We cannot afford to have these conversations conducted within unrealistic time constraints that do not allow for a fair and faithful determination that this is the right procedure for this person at this time. Third, we need to take steps to ensure that decision-making around MAID is not driven by institutional suffering that occurs as a result of a lack of good advance care planning. Finally, we need to develop further evidence and best practices about ameliorating the suffering that occurs around a MAID process. Much work has been done to ensure that MAID follows a person-centered approach. However, the moral and political contentiousness of MAID has perhaps made us reluctant to reveal that it is not without its own characteristic sufferings.

## Conclusion

This study has provided a descriptive account of nurses’ experiences with suffering in the context of MAID. Nurses enter into the MAID process with clients highly sensitized to the suffering of living with chronic illness and the iatrogenic suffering that can occur in the treatment context. The conversations that are required are complex, nuanced, and focused on hearing a life trajectory in which the decision to pursue MAID constitutes an authentic choice. Such an approach requires time and expertise. Findings indicate the complexity of considering suffering as an eligibility criterion while accepting the suffering story without judgement. Finally, nurses described the sufferings they perceived that clients and families encountered as part of the preparation for MAID. Further research is required to better understand how to attend to institutional suffering and to the various kinds of suffering that arise in the context of new end of life options. Suffering in the context of MAID requires a substantively different approach on the part of healthcare providers than what is common within our treatment-oriented frameworks.

## Data Availability

The datasets used and/or analysed during the current study are available from the corresponding author on reasonable request.

## Abbreviations

MAID: Medical Assistance in Dying

## Footnotes

1 Carter at para. 64, referring to *New Brunswick (Minister of Health and Community Services) v. G. (J.)*, [1999] 3 S.C.R. 46, at para. 58; *Blencoe v. British Columbia (Human Rights Commission)*, 2000 SCC 44, [2000] 2 S.C.R. 307 at paras. 55–57; *Chaoull v Quebec (Attorney General)* 2005 SCC 35 at para. 43, and *Rodriguez v British Columbia (Attorney General)*. [1993] 3 S.C.R. 519 at 587–589.

2 *Carter* at para. 63, referring to *Blencoe v. British Columbia (Human Rights Commission)*, 2000 SCC 44, [2000] 2 S.C.R. 307, at para. 54.

3 *Carter*, at para 65.

4 Ibid., at para. 1.

5 Ibid., at para. 1.

6 Ibid.

7 Ibid., at para. 14.

8 *Carter*, Ibid., at para. 335.

9 Ibid., at paras. 795–98, 815, 837, and 843 cited by the Supreme Court in *Carter*, *supra* note 2, at para. 102.

10 Ibid., at para 131

11 Only the patient can determine the intolerability of their suffering, this is not a practitioner interpretation.
